# Capsazepine (CPZ) Inhibits TRPC6 Conductance and Is Protective in Adriamycin-Induced Nephropathy and Diabetic Glomerulopathy

**DOI:** 10.3390/cells12020271

**Published:** 2023-01-10

**Authors:** Henning Hagmann, Naghmeh Hassanzadeh Khayyat, Mahsa Matin, Cem Oezel, He Chen, Astrid Schauss, Christoph Schell, Thomas Benzing, Stuart Dryer, Paul T. Brinkkoetter

**Affiliations:** 1 Department II of Internal Medicine, University Hospital Cologne, Faculty of Medicine, University of Cologne, 50937 Cologne, Germany; 2 Center for Molecular Medicine Cologne, University Hospital Cologne, Faculty of Medicine, University of Cologne, 50931 Cologne, Germany; 3 Department of Biology and Biochemistry, University of Houston, Houston, TX 77204, USA; 4 Cologne Cluster of Excellence on Cellular Stress Responses in Ageing-Associated Diseases (CECAD), 50931 Cologne, Germany; 5 Institute of Surgical Pathology, Faculty of Medicine, University Medical Center Freiburg, 79106 Freiburg, Germany; 6 Systems Biology of Ageing Cologne (Sybacol), 50931 Cologne, Germany; 7 Department of Biomedical Sciences, Tilman J. Fertitta Family College of Medicine, University of Houston, Houston, TX 77204, USA

**Keywords:** glomerular disease, podocyte, proteinuria, oxidative stress, lipid peroxidation

## Abstract

Reactive oxygen species (ROS), which excessively arise in diabetes and systemic inflammatory diseases, modify cellular lipids and cellular lipid composition leading to altered biophysical properties of cellular membranes. The impact of lipid peroxidation on transmembrane signaling routes is not yet well studied. The canonical transient receptor potential channel 6 (TRPC6) is implicated in the pathogenesis of several forms of glomerular diseases. TRPC6 is sensitive to membrane stretch and relies on a distinct lipid environment. This study investigates the effect of oxidative alterations to plasma membrane lipids on TRPC6 activity and the function of the glomerular filter. Knockout of the anti-oxidative, lipid modifying enzyme paraoxonase 2 (PON2) leads to altered biophysical properties of glomerular epithelial cells, which are called podocytes. Cortical stiffness, quantified by atomic force microscopy, was largely increased in PON2-deficient cultured podocytes. PON2 deficiency markedly enhanced TRPC6 channel currents and channel recovery. Treatment with the amphiphilic substance capsazepine in micromolar doses reduced cortical stiffness and abrogated TRPC6 conductance. In in vivo studies, capsazepine reduced the glomerular phenotype in the model of adriamycin-induced nephropathy in PON2 knockout mice and wildtype littermates. In diabetic AKITA mice, the progression of albuminuria and diabetic kidney disease was delayed. In summary, we provide evidence that the modification of membrane characteristics affects TRPC6 signaling. These results could spur future research to investigate modification of the direct lipid environment of TRPC6 as a future therapeutic strategy in glomerular disease.

## 1. Introduction

In most parts of the world, diabetic kidney disease (DKD) is the most common cause of chronic kidney disease and need for chronic renal replacement therapy [1]. Between 30 and 40% of patients with diabetes mellitus show features of diabetic damage to the kidney filtering units, known as glomeruli [2]. The human kidney contains around one million of these capillary tufts carrying highly specialized pericytes, called podocytes. The glomerular filtration barrier consists of three layers: the glomerular endothelial cells, the glomerular basement membrane, and podocytes [3]. Podocytes are terminally differentiated cells and are unable to proliferate to compensate for podocyte loss. Primary and secondary cellular processes (foot processes) extend from podocyte cell bodies and interdigitate with neighboring foot processes to form a filtration slit of around 40 nm in width. The resulting junction is bridged by a thin structure of transmembrane proteins known as the slit diaphragm.

Kidney-specific manifestations of diabetic metabolic states primarily appear in podocytes, including cytoskeletal rearrangement, de-differentiation, and apoptosis or detachment manifested by foot process effacement and podocyte loss. In addition, the proliferation of mesangial cells, which serve as scaffold and immunologic gatekeepers in the glomerular tuft, is an early sign of diabetic glomerular damage. The molecular pathologic mechanism of podocyte damage and mesangial proliferation is complex and incompletely understood. Alterations in the production or degradation of reactive oxygen species (ROS) are critical in the development of diabetic damage in many tissues including the kidney [4]. In DKD, the accumulation of ROS has been recognized as an important factor linking altered metabolism to podocyte damage, endothelial dysfunction, and finally inflammation and fibrosis [5]. However, ROS are not per se detrimental in podocytes and there is evidence that ROS are essential for the function of intracellular signaling pathways of podocytes in DKD [6]. The impact of ROS-induced lipid peroxidation of membrane lipids on cellular biophysics and on the highly active transmembrane signaling routes in podocytes remains enigmatic.

Signaling via the non-selective cation channel TRPC6 in podocytes is crucial in genetic and sporadic glomerular disease [7,8]. The TRPC6 channel is activated by membrane stretch [9,10]. In addition, G-protein coupled receptors and NADPH oxidase (NOX)-activity trigger TRPC6 conductance [10,11,12]. The activation of TRPC6 after oxidative stress was attributed to the activation of tyrosine kinases such as Src kinase [13]. In addition, modifications of the local lipid milieu of the plasma membrane lead to TRPC6 activation [10,14,15]. Efforts to target TRPC6 pharmacologically have been hampered by a lack of specificity and poor bioavailability of the available candidate substances [16,17].

We have previously shown that the transmembrane anti-oxidative arylesterase paraoxonase 2 (PON2) largely affects TRPC6 channel activity in glomerular epithelial cells [18]. In the absence of PON2, the activation of TRPC6 was enhanced by about 8-fold as compared to control following membrane stretch. In addition, recovery after activation of the channel was prolonged to 40 min in PON2 deficiency compared to 5 min in control. In addition, PON2 knockout resulted in an accumulation of ceramide, sphingomyelin, and cholesterol in podocytes. For other cell types, such as neurons and macrophages, it is well established that oxidized membrane phospholipids induce the accumulation of ceramide and cholesterol [19,20,21]. Knowing that PON2 antagonizes lipid peroxidation in cellular membranes, we speculated that increased bilayer stiffness due to the accumulation of ceramide and sphingomyelin might be the root of altered TRPC6 conductance. Accordingly, altering membrane biophysics by the introduction of amphiphilic substances may prevent TRPC6 hyperactivity and reduce the glomerular phenotype of PON2 deficiency in the adriamycin-induced nephropathy model as well as glomerular damage in diabetic AKITA mice.

## 2. Material and Methods

### 2.1. Cell Culture and shRNA-Induced Knockdown of PON2

Murine podocytes (obtained from S. Shankland, University of Washington) were maintained as previously described [22]. The podocytes were seeded on plastic dishes coated with collagen I. The podocyte cell lines express the SV40 large T temperature-sensitive antigen. The cells are cultured and maintained at 33 °C (termed “undifferentiated” cells). Transferring the cells to 37 °C induces cell cycle arrest and differentiation. The cells were left at 37 °C for 10 days and are referred to as “differentiated” podocytes at this stage.

RNA interference experiments were performed as described previously [23]. In brief, short hairpin RNAs (shRNAs) against the murine PON2 3′-untranslated region were designed based on the prediction of publicly available prediction programs (RNAi Designer; Invitrogen, Waltham, MA, USA) and multiple shRNA sequences tested in luciferase assays as previously described [24]. Stable cell lines were generated by transduction with lentivirus generated from constructs in the pLenti6.3 backbone.

The following sequences were used:

mPON2 hp#1: 5′_TGCTGTTAAATTCCCTCAGAATTGGCGTTTTGGCCACTGACTGACGCCAATTCAGGGAATTTAA_3′

mPON2 hp#2: 5′_TGCTGATTACTAGGTCAGTACTGATGGTTTTGGCCACTGACTGACCATCAGTAGACCTAGTAAT_3′

The re-expression of murine PON2 in PON2-deficient murine podocytes was achieved by retroviral gene transfer using mPON2 coding sequence lacking the 3′UTR in pBabe vector.

The capsazepine treatment was performed at concentrations as indicated, either in buffer containing 2 mM calcium or calcium-free PBS as described in the text.

### 2.2. Immunofluorescence Staining

Immortalized heat-sensitive murine podocytes were grown on collagen I cover slips and differentiated for 10 days at 37 °C. The cells were fixed in 4% paraformaldehyde, permeabilized using 0.1% Triton X-100 in PBS, stained with Alexa Fluor 594 Phalloidin (Life Technologies, Carlsbad, CA, USA), and mounted in Prolong Gold antifade containing DAPI (Thermo Fisher Scientific, Waltham, MA, USA). The images were acquired using an LSM 710/Axiobserver Z1 confocal microscope operated by ZEN2009 software (Carl Zeiss, Jena, Germany).

### 2.3. Atomic Force Microscopy

For atomic force microscopy (AFM) measurements, differentiated mouse podocytes were seeded on 34 mm culture dishes (TPP, Trasadingen, Switzerland) and grown over 2 days at 37 °C. AFM imaging was performed using a NanoWizard IV (JPK Instruments, Berlin, Germany) mounted on an Axiovert 200 inverted light microscope (Carl Zeiss, Jena, Germany). The microscopes were equipped with a heated stage (37 °C) and cells were imaged in an HEPES-buffered medium.

The indentation measurements were performed with silicon nitride cantilevers (MLCT, Bruker, Camarillo, CA, USA) with a nominal force constant of 0.01 N/m. The areas of 2500 µm^2^ were scanned in the QI imaging mode to acquire 3600 independent force measurements per area. The cells were indented up to a force of 0.9 nN at a vertical speed of 18 µm/s. The determination of Young’s Modulus was performed using the JPK data analysis software. The fit parameters were set to the Hertzian model according to Sneddon. The cantilever was specified as a quadratic pyramid tip shape with a half-angle to the edge of 15°.

The image analysis and quantification of apical stiffness were performed using Fiji software. For the determination of apical cell stiffness, grey scale topography maps were analyzed using Fiji contour line macro to mark cells and identify ROIs. The ROIs were transferred to the associated force map to determine cell stiffness. The statistical analysis was performed as univariate analysis, defining a *p*-value of <0.05 as statistically significant.

### 2.4. Electrophysiology

Whole-cell recordings were created at room temperature (22 °C) from immortalized podocytes as described in detail previously [9,25,26] using fire-polished borosilicate glass microelectrodes (4–6 MΩ) and an Axopatch 1D amplifier (Molecular Devices, Foster City, CA, USA). The bath was perfused at a constant flow rate (0.3 mL/min) and TRPC6 channels were activated by a bath application of a hypotonic stretch solution as described previously [9]. Macroscopic currents were monitored during 2.5-s voltage ramps from −80 mV to +80 mV from a holding potential of −40 mV and currents at +80 mV were quantified for statistical analysis. We have previously shown that the stretch-evoked cation currents are eliminated by TRPC6 knockdown [9] and by application of agents such as SAR-7334 that selectively inhibit TRPC6 [27].

### 2.5. Surface Biotinylation Assay and Immunoblot

The surface biotinylation assays were performed as previously described [28]. In brief, HEK 293T cells were grown in 6-well plates and transiently transfected with a V5-tagged TRPC6 expression construct and treated with increasing doses of capsazepine as indicated. Thereafter, the experimental procedures were performed at 4 °C. The cells were incubated with 1 mg/mL NHS-S-S-Biotin (Thermo Fisher Scientific, Waltham, MA, USA), washed with buffer containing 100 mM glycine, and lysed in detergent buffer as previously described. The biotinylated protein was bound to streptavidin beads (Pierce, Rockford, IL, USA). After washing 3 times in detergent buffer, the precipitates and lysates were analyzed using SDS-PAGE employing a V5-specific antibody. The quantitative analysis was performed using densitometry with ImageJ Fiji software.

### 2.6. Animals Models

The PON2 knockout mice were obtained from Sven Horke, University of Mainz, Germany [29]. The AKITA diabetic mice were obtained from Jackson (The Jackson Laboratory, Bar Harbor, ME, USA) [30]. The mice were housed according to the standardized specific pathogen-free conditions in the University of Cologne animal facility. All the animal experiments were performed in accordance with the guidelines provided by the LANUV NRW (Landesamt für Natur, Umwelt und Verbraucherschutz Nordrhein-Westfalen/State Agency for Nature, Environment and Consumer Protection North Rhine-Westphalia, approval numbers: 2013.A375, 2019.A067). The adriamycin nephropathy was induced in 11-week old male mice. Anesthesia/analgesia was performed with isoflurane/buprenorphine. After a single intra-venous injection of adriamycin (15 mg/kg body weight), the animals were followed for up to seven weeks and urinary albumin excretion was analyzed longitudinally on days 0, 14, and 49 as the albumin/creatinine ratio from spot urine samples. The quantification of albuminuria was performed using commercial kits (mouse albumin ELISA kit, Bethyl Labs, Montgomery, TX, USA; and urine creatinine assay, Cayman Chemical, Ann Arbor, MI, USA). For the analysis of serum creatinine and serum urea, the concentration blood samples were centrifuged at 3000 rpm and 4 °C for 10 min; 100 µL serum was extracted from the supernatant and analyzed for creatinine and urea levels in the central laboratory of the University Hospital Cologne, Germany. The renal tissue was embedded in OCT compound (Sakura, Torrance, CA, USA) or snap frozen in liquid nitrogen and stored at minus 80 °C or fixed in 10% neutral buffered formalin for immunostaining. Capsazepine was solubilized in DMSO at a concentration of 9 millimolar. The mice received daily intra-peritoneal injections of capsazepine (22 mg/kg body weight) or equivalent volume of vehicle. Their body temperatures were monitored by measurement with a rectal probe once weekly. Urine samples were obtained and analyzed as described above. In the AKITA mice, the glucose serum levels were checked before the initiation of capsazepine treatment and at the end of the study to reconfirm diabetic state. The histologic analysis included samples stained with periodic acid-Schiff (PAS), trichrome (TC), and acid Fushin Orange G (AFOG) stain. The quantification of mesangial matrix expansion was performed according to the previously published mesangial score [31].

## 3. Results

### 3.1. Lipid Peroxidation Affects Cell Morphology and Cortical Biophysics of Podocytes

**Figure 1:**
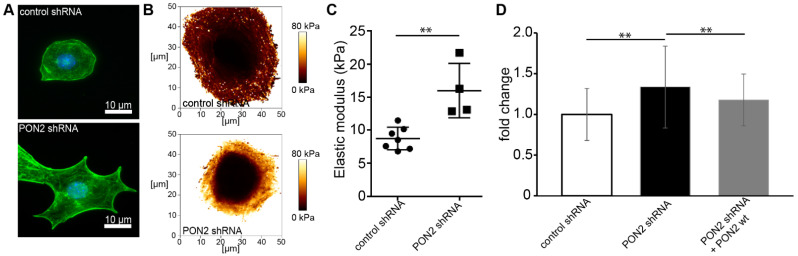
PON2 deficiency causes cytoskeletal rearrangement and increased cortical stiffness in podocytes. Phalloidin staining of stable podocyte cell lines expressing shPON2 or control shRNA (**A**). shPON2-expressing podocytes appear with pointed cell boundaries and pronounced cortical actin. Representative images of Young’s Modulus maps of PON2-proficient and PON2-deficient podocytes (**B**). Loss of PON2 results in increased cortical stiffness as compared to control (mean 15.99 vs. 8.75 kPa; *p* = 0.023; CI 95%: 3.327–11.14) (**C**). Force curves of podocytes expressing control shRNA (31 cells), PON2 shRNA (51 cells), or PON2 shRNA + PON2 wt (15 cells) of at least four independent experiments were analyzed for cortical stiffness relative to control shRNA. *y*-axis shows fold change of cortical stiffness compared to control shRNA expressing mouse podocytes. Cortical stiffness is increased in PON2-deficient podocytes (1.34-fold, SD 0.50 PON2 shRNA; ** *p* < 0.01). Increased cortical stiffness is reduced after stable re-expression of PON2 (1.17-fold, SD 0.32 PON2 shRNA + PON2 wt; ** *p* < 0.01) (**D**).

It is well known that glucose exposure induces ROS and leads to cytoskeletal rearrangement in podocytes [32,33,34]. Several studies reported increased cell stiffness in various cell types in diabetes [35,36,37]. Cytoskeletal architecture and cortical stiffness were assessed in differentiated stable mouse podocytes cell lines carrying lentiviral PON2 shRNA or control shRNA. The validation of PON2 knockdown and re-expression of PON2 wt is shown in Figure S1A. PON2-deficient podocytes show pronounced cortical actin distribution and pointed protrusions on cell margins as visualized by phalloidin staining (Figure 1A). Lower magnification views are provided in Figure S1B. Similar phenotypes can be observed in cultured podocytes treated with high glucose concentrations. To investigate cell stiffness of PON2-proficient and PON2-deficient podocytes, stable murine podocyte cell lines expressing PON2 specific shRNA or control shRNA were subjected to atomic force microscopy indentation measurements. Locally resolved stiffness maps show increased cell stiffness in PON2-deficient cells (Figure 1B,C). As compared to control shRNA expressing cells, PON2-deficient podocytes displayed increased cortical stiffness. The phenotype could be partially rescued by the stable re-expression of PON2 wildtype (Figure 1D).

### 3.2. Capsazepine Reverses Effects of PON2-deficiency on Cell Morphology and Cortical Stiffness

A higher content of ceramide in biological membranes, as seen in PON2-deficiency, renders the plasma membrane more rigid [18,38]. We hypothesized that such alterations of plasma membrane biomechanics could explain the aggravated effect of PON2 knockout on TRPC6-mediated podocyte injury, which we reported in a previous study [18]. Reversing altered biomechanics should result in reduced TRPC6 channel conductance and an ameliorated disease phenotype in mice.

Amphiphilic substances affect biophysical properties of the plasma membrane and exhibit membrane activity at micro- or millimolar concentrations. For example, capsaicin [N-(4-hydroxy-3-methoxybenzyl)-8-methylnon-6-eneamide] functions as a specific activator of TRPV1 channels at submicromolar concentrations but affects a plethora of membrane proteins when applied in micro- or millimolar concentrations [39]. Capsazepine [N-[2-(4-chlorophenyl)ethyl]-1,3,4,5-tetrahydro-7,8-di-hydroxy-2-H-2-benzazepine-2-carbothioamid] is an antagonist of capsaicin at submicromolar concentrations, blocking TRPV1 activation while acting synergistically with capsaicin in micromolar concentrations on various membrane channel proteins. This phenomenon is thought to be due to the amphiphilic structure of capsaicin and capsazepine. At micromolar concentrations, both molecules intercalate into the lipid bilayer, macerating and subsequently softening the plasma membrane [40,41].

In the next set of experiments, we employed capsazepine, a substance that passed through phase I clinical testing as pain medication, to assess the reversal of the PON2-deficiency phenotype in podocyte cell culture and animal models.

**Figure 2:**
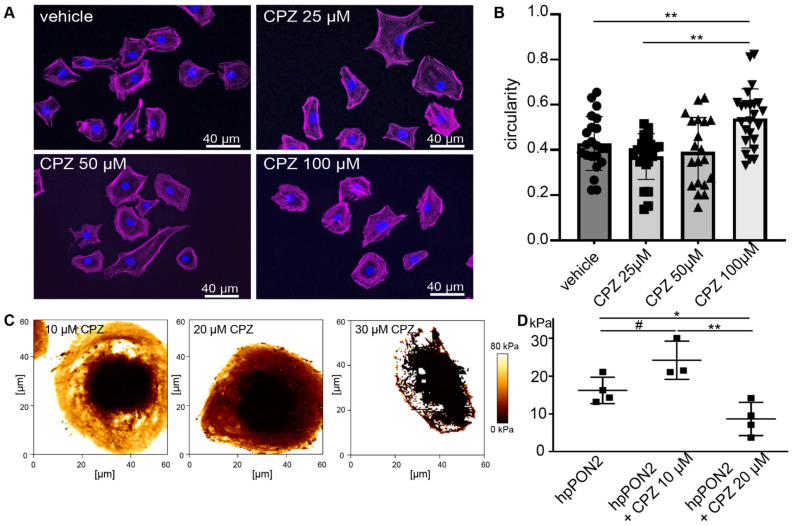
Capsazepine treatment reverses effects of PON2 deficiency. Phalloidin staining of stable podocyte cell lines expressing shPON2 after treatment with varying doses as indicated (**A**). Capsazepine (CPZ) treatment results in the reorganization of the actin cytoskeleton and a reversion to round morphology. Quantification of circularity of cells employing Fiji software (means 0.43; 0.37; 0.54 for vehicle, 25 µM, and 100µM, respectively; ** *p* < 0.01) (**B**). Representative images of Young’s Modulus maps of PON2-proficient and PON2-deficient podocytes after treatment with capsazepine (**C**). Cortical stiffness is reduced in a dose dependent effect (means: vehicle 16.26 kPa; 10µM CPZ: 24.23 kPa; 20 µM CPZ: 8.67 kPa; * *p* < 0.05; ** *p* < 0.01; # = ns) (**D**).

Differentiated PON2-proficient and deficient mouse podocyte were treated with increasing doses of capsazepine and cell morphology was observed following phalloidin staining (Figure 2A). At capsazepine doses of 100 µM, the cultured podocytes of both groups displayed transition from the starlet morphology in PON2-knockdown to rounded cell shapes (quantification in Figure 2B). When treated in calcium-free PBS, the effects on cell morphology were noted at concentrations of 20–30 µM (Figure S2). The cells expressing control shRNA showed no significant change in cell morphology.

To determine effects on cell stiffness, elastic modulus maps were generated by atomic force microscopy (AFM) imaging after pre-treatment with various doses of capsazepine. The addition of capsazepine showed a dose-dependent effect on cellular stiffness (Figure 2C,D) already with 20 µM capsazepine reducing cellular stiffness to baseline levels noted previously in PON2-proficient cells. Notably, the softening of the plasma membrane was so pronounced at capsazepine concentrations of 30 µM that the cells did not sustain the mechanical stress of force curve acquisition.

### 3.3. Capsazepine Treatment Completely Abrogates TRPC6 Response to Membrane Stretch

**Figure 3:**
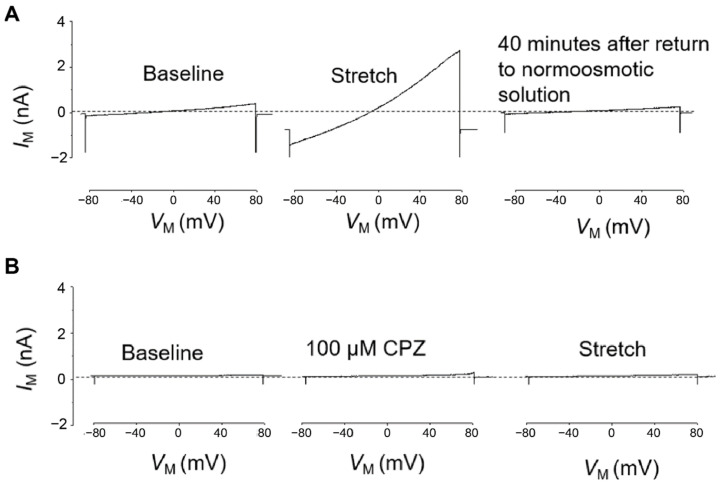
Hypotonic stretch-induced TRPC6 currents are largely enhanced in PON2 deficiency and reduced after capsazepine treatment. Representative whole cell voltage clamp recordings of PON2-deficient podocytes after treatment with 1% DMSO (**A**) or capsazepine (**B**). (**A**) In DMSO-treated PON2-deficient podocytes perfusion with a 70% hypotonic solution induced a marked increase in cationic currents recorded during application of ramp voltage commands (from −80 to +80 mV). These currents remained elevated for ~40 min after the cells were returned to a normotonic extracellular solution. (**B**) In a different PON2-deficient podocyte, exposure to 100 µM capsazepine in a normotonic solution caused a slight increase in currents recorded during the application of ramp voltage commands. No additional increase in currents was observed when cells were subsequently exposed to a hypotonic stretch solution.

TRPC6 activity was tested in whole-cell voltage clamp experiments on differentiated stable PON2-deficient or control mouse podocytes. PON2 knockdown cells treated with DMSO 1% (vehicle) showed strong response to membrane stretch as previously reported [18] (Figure 3A), which consisted of enhanced cation flow after immersion in hypotonic solution and prolonged recovery of around 40 min after changing the milieu back to isotonic solution. In contrast, the stretch activation of TRPC6 channels in PON2-deficient podocytes was abrogated after treatment with capsazepine (Figure 3B). Notably, capsazepine exposure led to a small activating current in control and in PON2-deficient podocytes (Figure 3B).

**Figure 4:**
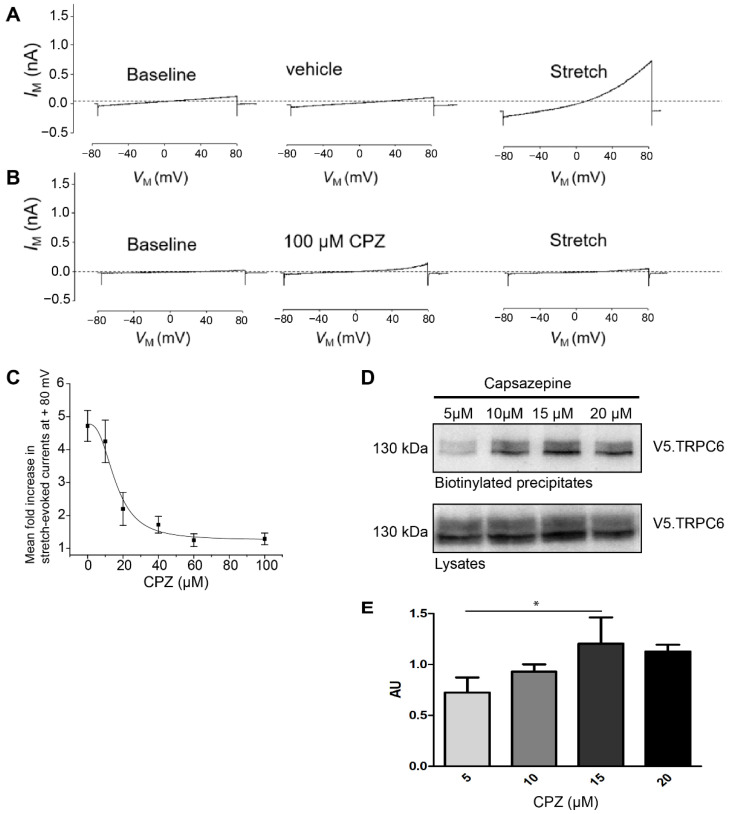
Stretch-induced TRPC6 currents are abrogated in PON2-proficient podocytes after capsazepine. Representative whole-cell voltage clamp recordings of control shRNA expressing podocytes. (**A**) Treatment with extracellular solution containing 1% DMSO had no effect on basal currents observed during ramp voltage commands. A subsequent hypotonic membrane stretch evoked a robust increase in cationic currents. (**B**) Treatment with 100 µM capsazepine results in small increases in cationic current but completely abrogated subsequent currents evoked by membrane stretch. (**C**) Capsazepine effects on wildtype podocytes are concentration-dependent with an ED50 of between 10 and 20 μM. (**D**) Membrane abundance of TRPC6 was quantified in surface biotinylation assays of V5.TRPC6 expressed in HEK293Tcells. Increasing doses of capsazepine resulted in the increased surface abundance of TRPC6. (**E**) Densitometric analysis revealed a significant increase in steady-state surface abundance of TRPC6 following capsazepine treatment (one-way ANOVA 0.023; AU 5 vs. 15 µM ttest * *p* < 0.05; *n* = 3). AU = arbitrary units.

The cultured mouse podocytes expressing control shRNA and treated with vehicle (DMSO 1%) showed regular activation of TRPC6 after application of hypotonic membrane stretch (Figure 4A). Additionally, in these cells, capsazepine abrogated the TRPC6 stretch response completely, whereas capsazepine by itself activated a small current (Figure 4B). The quantification of cation currents after incremental doses of capsazepine allowed to generate a dose response curve and to determine the half effective dose (ED50) for capsazepine (Figure 4C).

To test whether capsazepine affects TRPC6 membrane abundance, surface biotinylation experiments were performed after transient overexpression of V5-tagged TRPC6 in HEK293T cells and treatment with increasing doses of capsazepine (Figure 4D,E). A concentration-dependent increase in cell surface expression of TRPC6 was noted in the concentration range from 5 to 15 µM capsazepine (one-way ANOVA 0.023; AU 5 vs. 15 µM *p* < 0.05) (Figure 4D). At higher concentrations, e.g., 20 µM capsazepine, there was a trend toward reduced TRPC6 surface expression, but this effect did not reach statistical significance.

### 3.4. Capsazepine Reduces Proteinuria in the Adriamycin-induced Nephropathy Model

**Figure 5:**
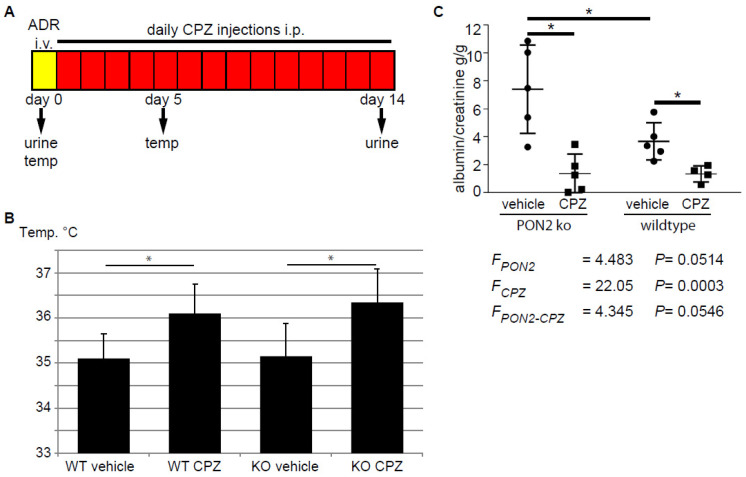
Daily administration of capsazepine reduces albuminuria in PON2-deficient and PON2-proficient mice during adriamycin-induced nephrosis. (**A**) Schematic representation of the in vivo protocol. After i.v. injection of adriamycin (ADR), mice were treated for 14 days with daily i.p. administration of capsazepine. Urine samples were collected on days 0 and 14 and body temperature was measured on days 0 and 5. (**B**) Body temperature was significantly increased in capsazepine-treated animals, measured after 5 days. Asterisks indicate * *p* < 0.05 by unpaired *t*-test. (**C**) Urine albumin/creatinine ratio at day 14 was significantly reduced in capsazepine-treated mice compared to vehicle-treated controls. This occurred in both PON2-proficient and PON2-deficient animals. Asterisks indicate * *p* < 0.05 by unpaired *t*-test. 2-way ANOVA shows differences of means for capsazepine treatment (F_CPZ_), a strong trend toward differences of means for PON2 (F_PON2_), and an interaction effect of both categorical variables (F_PON2-CPZ_).

Capsazepine has been investigated in clinical trials and is bioavailable after intra-venous injection and well tolerated. However, an increase in body temperature of around 1 degree Celsius in most of the individuals of the study population hampered the approval of the drug for clinical use [42]. To test the effect of capsazepine treatment in the setting of adriamycin-induced nephropathy in PON2 deficiency in vivo, we treated PON2-deficient mice as well as wildtype littermates with daily intraperitoneal injections of 20 mg/kg capsazepine or vehicle over a period of 14 days after induction of glomerular disease with adriamycin (Figure 5A). Mice receiving capsazepine exhibited an increase in body temperature of 1.0–1.2 degree Celsius compared to vehicle-treated controls without showing clinical signs of infection or malaise (Figure 5B). Most likely, increased body temperature indicates successful drug delivery and a systemic effect. Fourteen days after induction of adriamycin-induced nephropathy, spot urine samples were collected and the mice were sacrificed for histologic analysis. The quantification of the urine albumin/creatinine ratio revealed nephrotic range proteinuria in vehicle-treated PON2 knockout mice and less pronounced proteinuria in wildtype controls (7.41 g/g vs. 3.67 g/g). Treatment with capsazepine over 14 days significantly reduced proteinuria to 1.38 g/g in PON2 knockout mice (Figure 5C). Interestingly, proteinuria was also reduced in wildtype mice in adriamycin-induced nephropathy after capsazepine treatment to 1.35 g/g compared to vehicle. The statistical analysis with 2-way ANOVA showed clear differences of means with regard to capsazepine treatment and a strong trend toward an interaction effect of PON2 status and capsazepine exposure.

### 3.5. Capsazepine Ameliorates Glomerulopathy in the AKITA Diabetes Model

Translating the observation of cell stiffness-associated activation and recovery of TRPC6 and the potential therapeutic effects of reversal of cellular stiffness to another mouse model, we treated male diabetic AKITA mice with capsazepine starting at day 55 of life over a period of 45 days. AKITA mice develop insulin dependent diabetes after 3–4 weeks due to a mutation of the insulin 2 gene [43]. The animals received either 20 mg/kg capsazepine or vehicle (DMSO) by daily intraperitoneal injection. The urine samples were collected weekly and the mice were sacrificed for histologic and biochemical analysis on day 100 of life.

**Figure 6:**
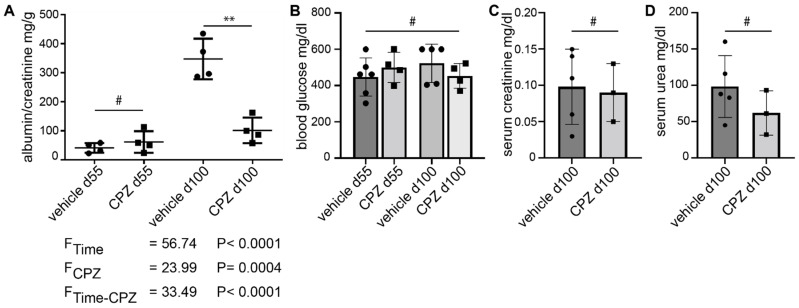
Daily administration of capsazepine over 45 days reduces albuminuria in type 1 diabetic AKITA mice. (**A**) At 55 days of age, AKITA mice do not show significant albuminuria. After 45 days of treatment (100 days of age), the albumin/creatinine ratio is reduced in capsazepine-treated AKITA mice (black squares) as compared to vehicle-treated littermates (black dots) ** *p*-value < 0.01; # not significant (ns)). 2-way ANOVA shows clear differences of means for age (F_Time_) and capsazepine treatment (*F_CPZ_*) and a clear interaction effect of both categorical variables (F_Time-CPZ_). (**B**) Blood glucose levels are elevated but are not affected by CPZ-treatment (means: 448.00 mg/dL vehicle d55; 500.25 mg/dL CPZ d55; 522.80 mg/dL vehicle d100; 453.25 mg/dL CPZ d100; # = ns). (**C**,**D**) Serum creatinine and serum urea levels show no significant differences between treatment groups at study termination (creatinine means: 0.098 mg/dL vehicle; 0.090 mg/dl CPZ; urea means: 98.4 mg/dL vehicle; 62.0 mg/dL CPZ; # = ns).

The albumin/creatinine ratio was significantly reduced in the capsazepine-treated animals as compared to vehicle-treated mice (102 mg/g vs. 347 mg/g; *p* < 0.01) (Figure 6A). The statistical analysis using 2-way ANOVA yielded clear differences of means for animal age (F_Time_) and capsazepine treatment (F_CPZ_), as well as a clear interaction effect of the two categorical variables. The blood glucose levels were increased in both groups at study entry and remained elevated without significant differences between groups until day 100 (Figure 6B). The serum creatinine and serum urea levels showed no significant differences between CPZ-treated and vehicle-treated animals at the end of the study (Figure 6C,D).

**Figure 7:**
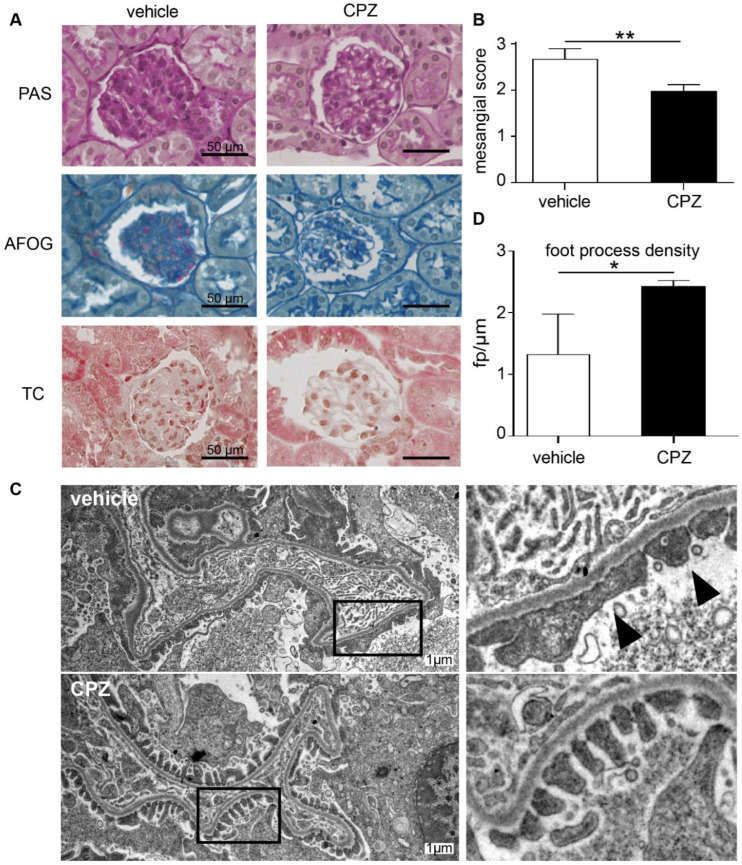
Capsazepine reduces glomerular damage in type 1 diabetic AKITA mice. (**A**) Histologic analysis of diabetic AKITA mice at day 100 of life after 45 days of capsazepine or vehicle treatment. Representative examples of PAS, AFOG, and trichrome staining of glomeruli from mice treated with vehicle (left column) or capsazepine (right column). Vehicle-treated mice show more glomerular damage compared to mice treated with capsazepine. (**B**) The mesangial score quantifying diabetic glomerular damage is significantly reduced in capsazepine-treated mice (means 2.67 vehicle vs. 1.98 CPZ; ** *p* < 0.01). (**C**) Representative electron microscopy images of kidney samples on day 100 show confined foot process blunting in vehicle-treated mice (arrow heads), whereas CPZ-treated mice show regular foot process morphology (black boxes in left column are regions enlarged in right column). (**D**) Quantification of number of foot processes per micrometer GBM shows higher foot process density for CPZ-treated mice as compared to vehicle controls (means: 1.33 fp/µm vehicle; 2.44 fp/µm CPZ-treated; * *p* < 0.05).

Periodic acid–Schiff (PAS), Acid Fushin Orange G (AFOG), and trichrome staining showed thickening of the basement membrane and mesangial cell as well as matrix expansion (Figure 7A). Based on the mesangial score [31], there was more severe diabetic glomerular damage in vehicle-treated mice as compared to samples from mice receiving capsazepine treatment (Figure 7B).

The electron microscopy of glomeruli of CPZ- and vehicle-treated animals showed activated endothelial and mesangial cells as expected for the AKITA model but no gross microstructural changes to glomerular cells or to the GBM. Notably, in both treatment groups as expected for low-level albuminuria, no expanded foot process effacement was observed (Figure 7C). However, the quantification of foot processes per micrometer GBM revealed higher foot process density in CPZ-treated animals as compared to vehicle controls (Figure 7D). This suggests blunting of podocyte foot processes in the vehicle-treated group, suggesting early impairment of podocyte function.

## 4. Discussion

Glomerular disease, especially in people with diabetes, is the leading cause of chronic kidney disease necessitating renal replacement therapy throughout the world [44]. There is a current unmet need for targeted therapy in pathological states that entail podocyte damage. In this study, we set out to find pharmacologic approaches to interfere with biophysical alterations of TRPC6 function that occur in the diabetic milieu.

The goal of this study was to investigate the effect of oxidative alterations to plasma membrane lipids on TRPC6 function and to understand how this affects TRPC6 function and ultimately the behavior of the glomerular filter. We have previously shown that PON2 counteracts the lipid peroxidation of membrane lipids [45]. Recently, we showed that PON2-deficiency in cultured podocytes and mouse kidneys increases cellular ceramide, sphingomyelin, and cholesterol content and largely affect TRPC6 function [18]. These alterations in lipid metabolism are well described in diabetes and are likely triggered by lipid peroxidation [19].

The evidence of a role for TRPC6 hyperactivity in diabetic kidney disease is evolving. Most commonly, TRPC6 channels are activated during G-protein coupled receptor (GPCR) cascades, such as angiotensin II (AngII) or adenosine triphosphate (ATP) bind to their specific receptors to activate Gq and downstream phospholipase C (PLC) to induce the generation of di-acylglycerols (DAG) in the plasma membrane [46]. Certain DAGs evoke an increase in the open probability of cell surface TRPC6 channels. Interestingly, the DAG effect is also present in excised membrane patches suggesting a mechanism of altered physical properties of the plasma membrane in direct vicinity to the TRPC6 channel protein [47]. In addition, TRPC6 is a redox-sensitive channel. The activation of NADPH oxidase 2 (NOX2) and NOX4 are essential for TRPC6 activation in response to AngII [12,48,49]. An indirect interaction of TRPC6 with NADPH oxidases suggests a role of local action of ROS, e.g., on the lipid environment of the channel protein via lipid peroxidation [11].

The paradigm of oxidative stress as a principle of the pathogenesis of diabetic complications was formulated in the early 1990s [50]. There are likely to be many sources of ROS in the diabetic milieu. However, it should be noted that specific types of reactive oxygen and nitrogen species have been implicated in normal cellular processes and the dogma of purely detrimental effects of mitochondria-derived ROS in diabetes has been challenged [51]. Moreover, there are many factors that appear to regulate TRPC6 activity in both normal and diabetic conditions [26]. Therefore, the separate consideration of different pathogenic mechanisms is warranted to unravel the molecular principles of diabetic kidney disease and find targets for therapy.

In addition to the effects on TRPC6 conductance, we observed that PON2 knockdown leads to morphological changes in podocytes with protuberant lamellipodia and pronounced cortical actin bundles, resembling the phenotype seen in hyperglycemic states [52]. Interestingly, this actin phenotype of subcortical bundles was linked to reduced cortical stiffness quantified by AFM in podocytes in a recent study [53]. In contrast, ceramides increase the molecular order of phospholipid membranes, leading to increased membrane rigidity [54]. In AFM measurements of cortical stiffness, control shRNA expressing podocytes yielded values of 8.75 kPa, which is in the same range as previously published [53]. Consistent with a stiffened plasma membrane due to the oxidative modification of lipids and increased ceramide content, cell stiffness of PON2- knockdown podocytes was largely increased. It is important to note that these kinds of indentation measurements cannot discriminate the contribution of the subcortical actin to cellular stiffness from effects due to plasma membrane composition, but in either case it is likely to affect the function of channels whose behavior can be altered, either directly or indirectly, by mechanical stimuli.

We employed the amphiphilic substance capsazepine to examine the possible role of increased plasma membrane rigidity in driving the effects of PON2 deficiency and in the diabetic milieu. Amphiphilic substances alter the biophysical properties of phospholipid bilayers by integrating into the membrane. The actions of capsazepine and its congeners are complex. Thus, capsazepine blocks the activation of TRPV1 at submicromolar concentrations but causes increases in membrane fluidity at higher concentrations [39]. In the present models of PON2 deficiency, treatment with capsazepine reversed the increased cortical stiffness and the morphological phenotype of cultured PON2 knockdown podocytes and completely inhibited TRPC6 function in PON2-deficient cells as well as in control and wildtype podocytes. The effects on morphologic phenotypes, cortical stiffness, and TRPC6 conductance were seen at different concentrations of capsazepine. Specifically, the effects on cortical stiffness were noted at capsazepine concentrations of 15–20 µM. The morphological changes depended on the calcium concentration of the medium during capsazepine treatment. When treated in calcium-free PBS, morphological changes were noted at much lower concentrations of the amphiphile.

Notably, since capsazepine is not thought to act through specific drug-receptor interactions, but rather by intercalation into the plasma membrane, high concentrations of the drug are needed. The exact mechanism of action and, e.g., effects on lipid phase transition are not well characterized. Several other studies focusing on capsazepine effects on TRP channels have used in high micromolar concentrations [55]. Another study reported an ED50 value for the inhibition of TRPM8 by capsazepine of 18 µM, which is similar to the ED50 of ~15 µM for TRPC6 obtained in our experiments [56].

Capsazepine has been previously employed in animal models to block TRP-channel activity. When blocking other channels than TRPV1, effective concentrations in the micromolar range were achieved by intraperitoneal injections [57,58]. Anti-convulsive effects were documented for capsazepine in even higher micromolar doses [59]. In experiments testing the in vivo effect of capsazepine in adriamycin-induced nephropathy and diabetic nephropathy, we employed capsazepine in a dose of 20 mg/kg body weight with daily intraperitoneal injections.

Adriamycin-induced nephropathy is a widely used model of glomerular disease triggered by free radical generation, oxidative stress, and lipid peroxidation, especially in rats [60]. However, in contrast to rats, adriamycin treatment does not induce robust histologic phenotypes in mice. The degree of damage in mice is strongly dependent on the genetic background and the histologic changes develop later and tend to be less consistent. We had previously titrated the dose of adriamycin in PON2 proficient and deficient mice in the CD1-background to induce nephrotic range proteinuria after 2 weeks with recovery in wildtype animals thereafter. Consistent with previous reports, the histologic changes were not remarkable in wildtype animals at 49 days after adriamycin treatment and were subtle in PON2 knockout animals [18]. Therefore, we did not include histologic analysis of PON2 knockout mice in the adriamycin model treated with capsazepine in this manuscript. However, adriamycin induces albuminuria which can be used as a readout in adriamycin-induced nephropathy mice treated with capsazepine.

Capsazepine treatment produced a robust protective effect in PON2 knockout mice in the adriamycin-induced nephropathy model, characterized by a reduction in albuminuria at 14 days. Interestingly, albuminuria was also reduced in wildtype mice after induction of adriamycin-induced nephropathy when treated with capsazepine over 14 days.

In diabetic kidney disease, TRPC6 signaling in podocytes is overactive due to increased humoral factors such as Ang II and others, as well as increased ROS production via NOX (reviewed in [61]). The prolonged excessive calcium load is detrimental to podocytes, finally resulting in podocyte loss and glomerular sclerosis. There are very few practical animal models that phenocopy human diabetic kidney disease. The available classical murine models of DKD such as the streptozotocin-induced and AKITA type 1 diabetes models or obese type 2 models do not develop all features of diabetic nephropathy in humans [62]. In diabetic patients, albuminuria and the expansion of the mesangial matrix are linked to DKD progression [63,64]. Focusing on these two readouts in AKITA type 1 diabetic mice, the effect of capsazepine on the development and progression of DKD was investigated. A weakness of the present study is that the sample size is small (N = 4 per group). Nevertheless, capsazepine treatment showed consistent effects, including reduced proteinuria and reduced mesangial pathology according to Gurley et al. [65]. The investigation of capsazepine-induced changes of cortical stiffness in cultured podocytes after treatment with high glucose was not successful because the cells did not sustain the mechanical manipulation during AFM measurements.

It is likely that the effect of capsazepine is unspecific and untargeted and that modifications of plasma membrane biophysics act on a plethora of membrane channels and receptor proteins. Indeed, the evidence presented here is correlative, as we do not know that the observed effects on TRPC6 explain the protective effects seen here. Nevertheless, the data presented herein provide evidence that the modification of membrane stiffness influence TRPC6 signaling and may represent a therapeutic approach for DKD and potentially other glomerular diseases in the future.

## Data Availability

Data are available upon request to corresponding authors.

## Supplementary Materials

The following supporting information can be downloaded at: https://www.mdpi.com/article/10.3390/cells12020271/s1, Figure S1: Validation of PON2 knockdown and PON2 re-expression in podocyte cell lines; Low magnification images phalloidin staining of murine podocytes +/− PON2; Figure S2: Phalloidin staining of PON2-proficient and deficient podocytes after treatment with varying doses of capsazepine in calcium-free PBS.
