# Supplementary material for: Capsazepine (CPZ) Inhibits TRPC6 Conductance and Is Protective in Adriamycin-Induced Nephropathy and Diabetic Glomerulopathy
Source: Cells. 2023 Jan 10;12(2):271. doi: 10.3390/cells12020271 (PMC9856956; doi:10.3390/cells12020271)
Supplement: Supplementary file 1 [file cells-12-00271-s001.zip › cells-1986622-SI.pdf]

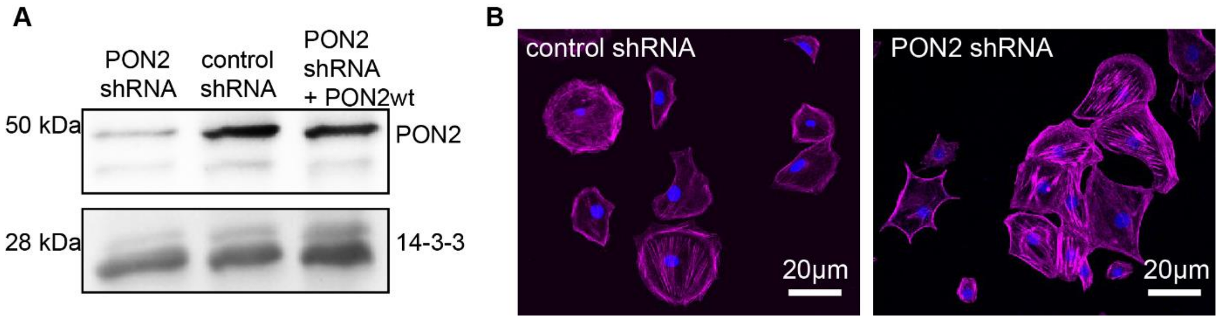

Figure S1. (A) Immunoblot analysis of lysates of stable murine podocyte cell lines expressing PON2-specific shRNA targeted to the 3′UTR of PON2 (PON2 shRNA), scrambled shRNA (control shRNA), and a murine podocyte cell line expressing PON2 shRNA and wildtype murine PON2 lacking the 3′UTR (PON2 shRNA + PON2wt). **(B)** Low magnification view of PON2-proficient and PON2-deficient mouse podocytes stained with phalloidin.

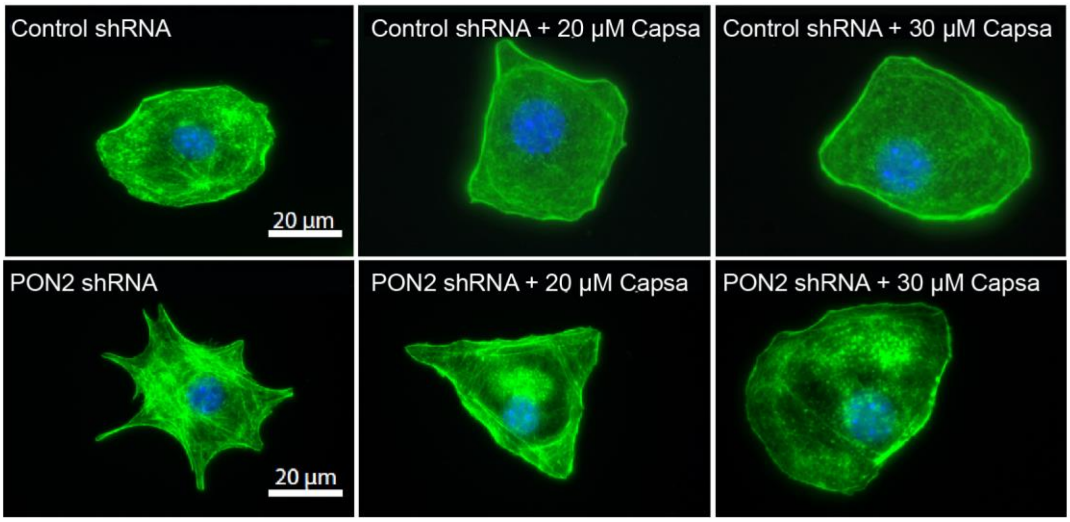

Figure S2. Phalloidin staining of PON2-proficient and –deficient podocytes after treatment with varying doses of capsazepine in calcium-free PBS. Capsazepine (Capsa) treatment results in reorganization of the actin cytoskeleton in PON2-deficient cells and reversion of the spiked phenotype to round morphology.
